# Recurrence and Complication Rates of Surgical Treatment for Blount’s Disease in Children: A Systematic Review and Meta-Analysis

**DOI:** 10.3390/jcm12206495

**Published:** 2023-10-12

**Authors:** Marco Ramella, Alessandro Depaoli, Grazia Chiara Menozzi, Giovanni Gallone, Tosca Cerasoli, Gino Rocca, Giovanni Trisolino

**Affiliations:** Unit of Pediatric Orthopedics and Traumatology, IRCCS Istituto Ortopedico Rizzoli, 40136 Bologna, Italy; marco.ramella@ior.it (M.R.); alessandro.depaoli@ior.it (A.D.);

**Keywords:** Blount’s disease, infantile tibia vara, late onset tibia vara, surgery, osteotomy, hemiepiphysiodesis, external fixator

## Abstract

Background: Blount’s disease is a growth disorder of the proximal tibia that causes progressive genu varum in children. Surgical treatment is recommended if the deformity worsens, but which intervention is best remains controversial. This study aims to identify factors influencing outcomes and determine the most effective surgical approach. Methods: A systematic review was conducted of studies published before January 2022. Results: In total, 63 retrospective studies with CEBM IIIb/IV levels were included (1672 knees in 1234 patients). The most commonly reported treatment was acute correction via osteotomy (47%), followed by hemiepiphysiodesis (22%) and gradual correction (18%). Combined procedures were reported in 13% of cases. The overall recurrence rate was 18%, with a significant difference when comparing the recurrence rates after gradual correction with those after hemiepiphysiodesis (7% and 29%, respectively). Major complications beyond recurrence were observed in 5% of cases. A meta-analysis of the available raw data showed a significantly increased recurrence rate (39%) among treated children who were between 4.5 and 11.25 years of age and were followed for a minimum follow-up of 2.5 years. Conclusions: Overall, poor evidence with which to establish an optimal treatment for Blount’s disease was found. This study remarked on the need for early diagnosis, classification, and treatment of infantile tibia vara, since a significant rate of recurrence was found in neglected cases.

## 1. Introduction

Blount’s disease is a growth disorder of the proximal medial portion of the tibia that causes progressive genu varum in children and adolescents [1,2]. The main clinical sign is a varus deformity in the proximal tibia, which may be progressively associated with other deformities, including intratorsion of the tibia, knee procurvatum, leg length discrepancy, valgus of the distal tibia, and varus or valgus of the distal femur [3]. Although the exact cause of Blount’s disease is unknown, obese children, those of Afro-Caribbean descent, and early walkers are more prone to develop the disease [2]. There is general agreement that Blount’s disease should be distinguished into two main clinical forms, based on the age of onset: infantile tibia vara (ITV) and late-onset tibia vara (LOTV) [2,3,4]. ITV usually occurs in children between 2 and 5 years of age, and it is bilateral in 50% of cases. LOTV is rarer, typically unilateral, less severe, and usually affects adolescents older than 10 years of age. The diagnosis is based on physical examination, radiographic evaluation, and monitoring of the progression of the deformity over time [2].

Surgical treatment is recommended when genu varum progresses, with the goal of realigning the knee axis and arresting the progression of the deformity [2,5]. Several surgical strategies have been recommended, including various types of osteotomies, progressive correction with an external fixator, temporary lateral hemiepiphysiodesis, and chondrodiastasis [2,3,4,5,6,7]. Currently, there is insufficient evidence with which to determine the most effective treatment approach for managing Blount’s disease in children, and there is also uncertainty regarding the factors that may influence treatment outcomes.

The objective of this study was to conduct a systematic review and meta-analysis of the published research on the surgical treatment of Blount’s disease. The primary goal was to identify factors that may influence treatment outcomes, with a particular focus on determining the most effective surgical option for minimizing the recurrence and complication rates.

## 2. Materials and Methods

### 2.1. Bibliographic Research

This systematic review adhered to the guidelines outlined in the PRISMA 2020 statement [8]. The protocol was registered with the international prospective register of systematic reviews (PROSPERO CRD42023465156). One electronic literature search of the Ovid, PubMed, Embase, and Cochrane Library databases for the term “Blount” was conducted on 18 January 2022 by one author (G.G.), and then replicated by adding the appropriate MeSH terms. The search was not restricted in terms of year of publication, journal type, or level of evidence. In addition, the bibliographies of all selected articles were checked to include any additional relevant studies.

### 2.2. Inclusion and Exclusion Criteria

Articles were evaluated according to the following inclusion criteria: (1) original articles on Blount’s disease, (2) written in English, (3) discussing surgical treatment, (4) of three or more patients who were (5) younger than 18 years of age, and (6) peer-reviewed. The following exclusion criteria were also applied: (1) articles that did not report an original case history (e.g., reviews, expert opinions, and surgical technique manuals), (2) posters, conference abstracts, or thesis work that did not have a corresponding peer-reviewed published article, (3) articles written only in languages other than English, (4) studies that did not clearly illustrate the surgical technique applied and/or that reported only conservative treatments, (5) case series including only adult patients, (6) or those including fewer than three patients affected by Blount’s disease. Furthermore, articles including fewer than three patients and case series reporting data not comparable with others—thus, not suitable for review and meta-analysis—were evaluated for recurrence and serious complications to avoid overestimation of the positive results of studies included in the present review.

### 2.3. Selection of Articles

Two authors (M.R. and A.D.) independently conducted the initial selection by reading the titles and abstracts of all articles found. Then, articles that met all criteria, or those that could not be excluded with certainty, were retrieved and evaluated for data extraction. If there was disagreement between the reviewers as to whether an article should be included, a pediatric orthopedic specialist (G.T.) was consulted to decide.

### 2.4. Methodological Quality and Risk of Bias Assessment

The articles’ levels of evidence were determined according to the Oxford Centre for Evidence-Based Medicine (CEBM) criteria, while the quality levels of the case series were evaluated with the modified Coleman Methodology Score (mCMS) and the Methodology Index for Non-Randomized Studies (MINORS) [9,10,11].

### 2.5. Data Extraction

From the selected articles, the following data were extracted and entered into an Excel table (Microsoft, Redmond, Washington DC, USA): first author; year of publication; state; patient attributes, including demographic information (sex, laterality, ethnicity, age at diagnosis, and family history), obesity (categorized as a Body Mass Index over the 95th percentile for age and sex), and preoperative and postoperative clinical data (comorbidities, previous surgical history, clinical deformity, presence and degree of tibial intratorsion, pre- and postoperative symptoms, and outcome scores); radiographic features (stage according to Langenskiöld and/or Laville for ITV, and angles and biometric measurements reported by the authors); aspects related to the surgical treatment (age at surgery, type of treatment, associated procedures, time of immobilization, time for consolidation, fixation technique, and follow-up); recurrence; complications; and need for further surgical procedures.

Each surgical procedure was evaluated according to (1) the number of tibial and femoral osteotomies and/or corticotomies performed; (2) the types of corrections planned (medial plate elevation, angular correction, rotational correction, lengthening of the limb), and specifying if (3) correction was achieved with acute correction (AC) or gradual correction (GC); (4) the association of proximal lateral tibial hemiepiphysiodesis (hE); (5) the type of fixation (no fixation or cast, an external fixator, pins, Blount staples, plates); and (6) the type of bone graft. According to those variables, surgical procedures were categorized into six main groups:

- gradual correction of at least one of the deformities (GC);
- gradual correction of at least one of the deformities, combined with hemiepiphysiodesis (GC + hE);
- acute correction of all deformities (AC);
- acute correction of all deformities, combined with hemiepiphysiodesis (AC + hE);
- isolated growth modulation via hemiepiphysiodesis (hE);
- hemichondrodiastasis (hChD).

Complications were divided into mild and severe, as follows: the former included conditions that delayed functional recovery insignificantly (e.g., superficial infections, including pin tract infections, or hypertrophic scars); on the other hand, conditions that consistently impacted functional recovery and/or required additional surgery (e.g., fixation system failures, deep infections, neurological deficits, and overcorrections) were considered severe. To compare results, complications were evaluated according to the Clavien–Dindo–Sink classification, as modified by Dodwell et al. (mCDS) [12]. Complications up to grade 2 were categorized as minor, while complications graded 3 to 5 were classified as major.

### 2.6. Statistical Analysis

Nonparametric statistical analysis methods were applied to assess correlations and associations, depending on the nature of the variables. In comparisons of means and prevalences of outcome data, the heterogeneity of nonparametric variables was checked with Cochrane’s Q test, considering a heterogeneity of less than 25% (I^2^ < 0.25) as acceptable [13]. The double arcsine transform, according to Freeman–Tukey, was applied to stabilize variance before the interpolation of data [14]. Univariate and multivariate analysis with Bonferroni correction were applied to test factors impacting the rates of recurrence and complications. Contingency tables were compiled to estimate the influence of preoperative and intraoperative variables on the recurrence rate.

Studies that reported raw data were further analyzed to perform a meta-analysis of homogeneous data. After visual analysis screening, the STATA threshold regression model, choosing an optimal number of thresholds using the Bayesian information criterion, was used to calculate the number and the values of thresholds. Subsequently, multivariate analysis was conducted to assess potential correlations between variables.

## 3. Results

### 3.1. Characteristics and Methodological Qualities of the Included Studies

**Figure 1:**
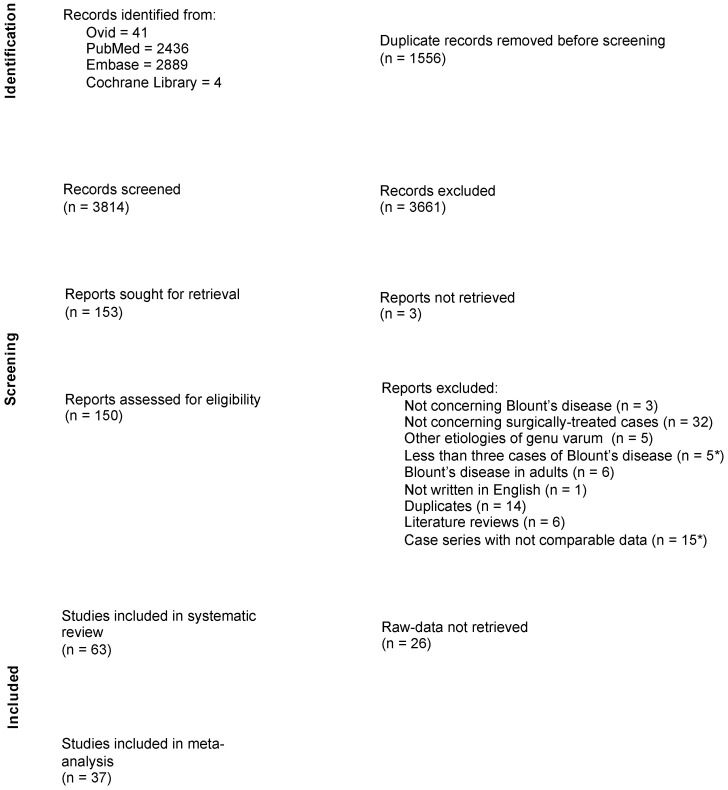
Flow chart of article selection process. (*)—these articles, and the other nine articles already excluded through the reading of abstracts (total 29), were further evaluated for the screening of recurrence and major complications to avoid an overestimation of positive results in the final analysis. This diagram was made according to the PRISMA 2020 statement guidilines [8].

**Figure 2:**
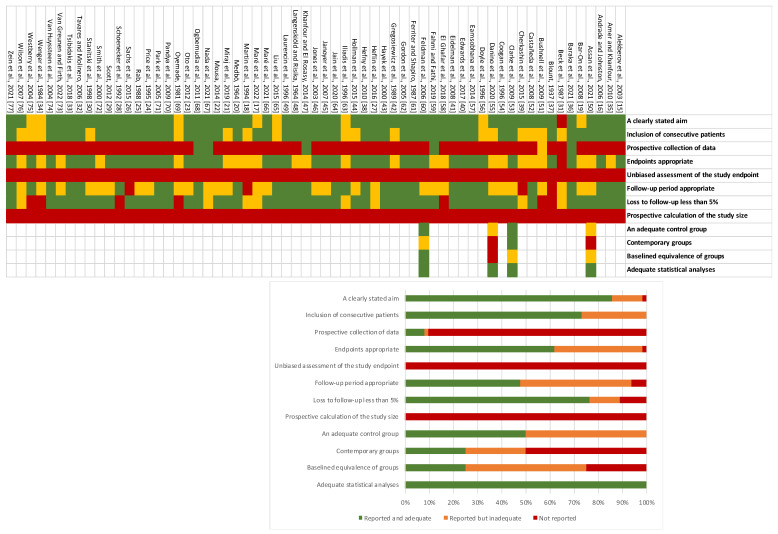
Results of MINORS analysis for bias in individual studies. In the upper image, a green mark indicates that the domain was adequately addressed; a yellow mark indicates that the domain was inadequately addressed; a red mark indicates that the domain was not addressed. The proportions of eligible studies with adequate, inadequate, and unreported data for each MINORS domain are depicted in the lower image.

The systematic review included 63 studies out of 3814 retrieved records (1672 knees in 1234 patients; see Figure 1), spanning an overall period of 84 years (1937–2022). Studies included an average number of 20 patients (range 3–59). Four studies were retrospective comparative studies (CEBM level 3b), while the remaining studies were retrospective case series (CEBM level 4). The mean mCMS was 43.7 (range 10–77), while the mean MINORS scores were 13.5/24 for CEBM level 3b studies (range 10–16) and 8.4/16 for CEBM level 4 studies (range 3–12; see details in Table 1). No significant improvement was observed in the quality of studies and reports across the years (*p* > 0.12). The risk of bias assessment according to the MINORS tool has been performed (see details in Figure 2). Thirty-five studies reported raw data about 676 knees, allowing for meta-regression analysis [15,16,17,18,19,20,21,22,23,24,25,26,27,28,29,30,31,32,33,34,35,36,37,38,39,40,41,42,43,44,45,46,47,48,49]. Out of these cases, authors explicitly specified the diagnosis as either ITV or LOTV in 349 patients (472 knees), with ITV being the more prevalent condition (67%; C.I. 95% = 63–72%).

### 3.2. Demographics, Clinical, and Radiographic Characteristics

Studies were conducted in centers in Africa (16 studies), Asia (7 studies), Europe (10 studies), and North America (28 studies). Patients from different continents were included in two case series (see details in Supplementary Materials, Table S1) [50,55]. Sex was reported for 975 patients (483 females, M/F ratio = 1.02/1). The sex distribution among ages was not homogeneous (see Supplementary Materials, Figure S1), with ITV being more frequent among females (68%; 95% C.I. = 63–74%), while LOTV was more frequent among males (69%; 95% C.I. = 60–77%; *p* = 0.0001). A total of 433 patients (35%) had bilateral involvement. Bilaterality was more common among ITV cases (38% in ITV vs. 28% in LOTV), although this difference did not reach statistical significance (*p* > 0.05).

Information about BMI was available in 398 cases, showing obesity in 56% of cases. Clear information about ethnicity was available in 9% of cases.

Six studies did not report any radiographic preoperative assessment of the deformities [23,24,28,37,41,44]. In the remaining studies, there was consistent variability in the radiographic assessments of the deformities (see details for articles with raw data in Supplementary Materials, Table S2). Twenty-nine studies reported raw data about preoperative radiographic evaluation. The most reported radiographic parameter was the anatomical tibiofemoral angle (aTFA) [15,18,19,22,25,26,29,32,40,43,48,49], while only 264 knees were rated according to the Langenskiöld classification [16,18,19,22,25,26,27,29,32,35,38,42,43,45,46,48,49]. Usage of the Langenskiöld classification showed a slight correlation with age at treatment (beta-coefficient = 0.25; 95% C.I. = 0.19–0.30; *p*-value = 0.0001).

### 3.3. Surgical Outcomes, Recurrence, and Complications

The types of surgical procedures utilized are reported in Table 2. The most reported treatment was acute correction with osteotomy (AC, 47%), followed by hemiepiphysiodesis (hE, 22%) and gradual correction (GC, 18%). Combined procedures (AC + hE and GC + hE) accounted for 13% of treated knees. Hemichondrodiastasis (hChD) was reported in just one study (four knees), showing unacceptable rates of recurrence and complications, so it was excluded from further pooled data analysis.

Recurrence was reported in 60 studies (1579 knees, 95% of the entire pool). The average recurrence rate was 18% (C.I. 95% = 14–22%, I^2^ = 0.22), and was higher among hE patients (29%) and lower among AC + hE patients (5%). The recurrence rate was significantly lower in the GC group compared with that of the hE group (*p* = 0.03). No difference was found when comparing all other groups.

The 16 studies published before the year 2000 reported an overall prevalence of recurrence of 25%, with good homogeneity (95% I.C. = 16–36%; I^2^ = 0.22), while the remaining 43 studies, published after 2000, showed a prevalence of 17%, with a more scattered distribution (C.I. 95% = 12–22%; I^2^ = 0.32). The difference was not statistically significant (*p* = 0.10).

A meta-regression of the raw data showed that the recurrence rate was higher in Caucasians compared to in Africans (27.3% vs. 2.1%; *p* = 0.018), and in obese patients compared to normal-weight patients (29.4% vs. 12.5%; *p* = 0.001), while no significant associations emerged regarding sex, side of deformity, or history of previous corrective knee surgery. Among radiographic measures, only preoperative femoral condyle–tibial shaft angle (FC-TS angle) showed some association with the risk of recurrence, which was measured in just 26 knees from three studies (*p*-value = 0.013; see Table S1 of Supplementary Materials) [18,21,35].

The mean follow-up duration and recurrence rates were concurrently available in 54 studies (1373 knees, 82% of the entire pool), demonstrating that patients with recurrence had a significantly longer follow-up period (6.7 ± 4.0 years) compared to patients without recurrence (5.5 ± 3.7 years; *p* = 0.02). The threshold regression model for recurrence by follow-up found the highest prevalence of recurrence among patients with a follow-up between 1.7 and 2.5 years (46% with 95% I.C. 36–58%; *p*-value = 0.0001; see details in Supplementary Materials, Table S2).

Complications were specified in 56 studies (1363 knees, 82% of the entire pool). The overall rate of minor complications was 18%, while that of major complications was 5% (see Table 2). The screening of case series and case reports not included in this review estimated comparable values of recurrence (17%) and major complications (5%) in a further 345 knees from 260 patients.

No cases of limb amputation or fatalities resulting from disease-related complications were documented in the studies. Minor complications were significantly more frequent in GC (49%) and GC + hE (74%) patients when compared with other groups (*p*-value = 0.0001). However, apart from AC + hE (I^2^ = 0.12), all surgical groups showed high heterogeneity in the rate of minor complications (I^2^ between 0.42 and 0.57). There was no statistically significant difference in the rate of major complications among the groups.

**Figure 3:**
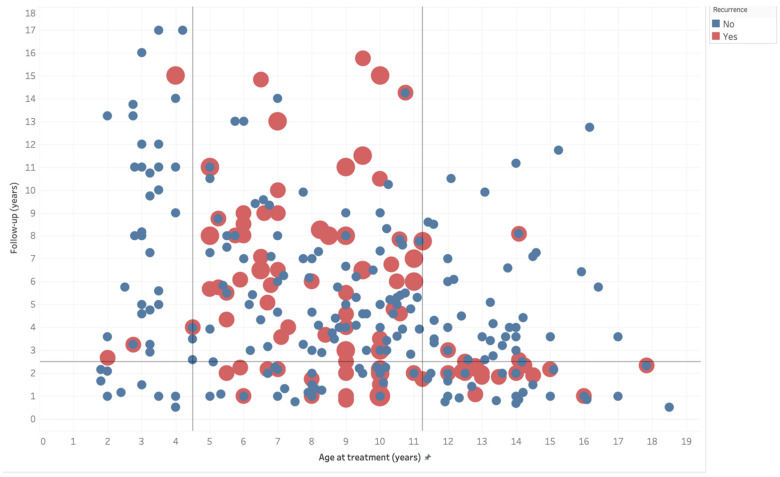
Distribution of 298 knees by age at treatment (*x*-axis in years) and follow-up duration (*y*-axis in years), with red markers indicating recurrences. Thresholds found with a regression model were marked as lines, highlighting a conspicuous prevalence of recurrences in patients treated between 4.5 and 11.25 years of age with at least 2.5 years of follow-up.

Upon visual analysis of the raw data, we observed a non-uniform pattern in recurrence distribution concerning the age at treatment and the follow-up duration. Specifically, a higher prevalence of recurrences was noted in patients treated between 4.5 and 11.25 years of age and followed for at least 2.5 years (see Figure 3). Within this subgroup (169 knees derived from 13 different studies; see Table S3 in Supplementary Materials), the recurrence rate was 39%, or up to 58%, when considering the largest group of cases, those treated with AC alone. However, 21 cases treated with combined procedures showed no recurrence.

Surgical procedures performed in patients treated before 4.5 years of age were acute osteotomies in most cases, with a low recurrence rate (7%; see details in Table S4 of Supplementary Materials). Surgical procedures performed in patients over 11.25 years of age were heterogeneous (see details in Table S5 of Supplementary Materials). With the numbers available, patients in the hE group showed a 32% recurrence rate, which is significantly higher than that of the GC group (*p* = 0.014), while patients treated with AC had a recurrence rate comparable with patients treated with GC (*p* = 0.99) and hE (*p* = 0.28).

## 4. Discussion

Several systematic reviews have been conducted on Blount’s disease. However, this study represents the most comprehensive systematic review, comparing the results of all reported surgical treatments for Blount’s disease in children, largely encompassing previous systematic reviews on the same topic, which each investigated between 4 and 32 studies [1,78,79,80,81,82,83]. This study is the first systematic review that compared the results of all surgical methods applied in the treatment of Blount’s disease. This approach, despite being a possible confounding factor, appears increasingly necessary for this condition, due to the increasing use of combined techniques.

Two systematic reviews were focused on epidemiology and risk factors for Blount’s disease [1,81]. Gilbody et al. compared the results of AC and GC among 18 studies (1 comparative), finding weak evidence for AC over GC [78]. Phedy and Siregar compared various osteotomy techniques and fixation methods among four studies, and did not report any conclusion based on their statistical analysis of the data, but recommended surgery tailored according to the patient’s age and the surgeon’s skills [79]. Sananta et al., by reviewing 15 studies and only focusing on osteotomies, concluded that, despite the unpredictable results, most authors recommended corrective osteotomy, preferably before age 4, in line with our findings [83]. Moreover, they suggested applying other techniques according to deformity severity, but found no significant benefit in combined techniques [83]. Burghardt et al. conducted a narrative review of twelve studies about hemiepiphysiodesis for the treatment of Blount’s disease, concluding that the isolated use of this procedure may lead to poorly predictable results and frequent under-correction [80]. Another systematic review by Jain et al. confirmed this observation, reporting an overall recurrence rate of 49% among eight case series of patients treated with hemiepiphysiodesis [64].

The aim of this wide review was not only to confirm the variation in recurrence rate at different ages of treatment, especially for ITV, but to verify whether there are effective surgical techniques for patients with higher risks of recurrence. Our findings confirmed the generally low quality of reports, which can be attributed to the disease’s rarity, the extensive timeframe encompassing the studies included, and the diverse array of treatment approaches, as well as the comprehensive assessment of pre-operative clinical and radiographic variables, reported outcomes, and length of follow-up. Nevertheless, our research yields valuable insights that can enhance future investigations into this disease.

### 4.1. Definition of ITV/LOTV Subgroups

The recognition of “early onset” and “late onset” presentations of Blount’s disease has been evident since the initial case series reported in the literature [23,28,44]. However, our investigation revealed notable disparities among authors and inconsistent reporting in the criteria used to delineate the ITV/LOTV subgroups. We confirmed that ITV and LOTV have some epidemiological differences (e.g., sex distribution and prevalence of bilaterality) [1,2]. However, the primary differentiation between these conditions lies in their etiopathogenetic mechanisms, rendering them markedly dissimilar in their natural progressions. In the case of ITV, there is an early varus knee presentation that differs from the typical axis correction progression described by Salenius and Vankka [84]. Conversely, LOTV follows a different trajectory, characterized by normal knee development during childhood, followed by a late growth arrest of the tibial plateau around the transitional age (10–12 years), leading to a gradual varus deformity during pubertal development.

The main challenge is accurately classifying a varus knee during the intermediate stage, defined by some authors as the “juvenile presentation”, which typically occurs between ages 4 and 10 [55,85]. Distinguishing between a neglected form of ITV and an early onset of LOTV in this stage is difficult. Our systematic review highlighted a notably high recurrence rate in this group, particularly when cases were monitored over an appropriate duration. Our findings suggest that what many cases referred to as “juvenile” likely resulted from a missed diagnosis or delayed surgical intervention of a subtle and overlooked ITV, leading to a notable deterioration in the clinical condition [4].

### 4.2. Overall Treatment Prognosis

The predominant surgical technique applied in Blount’s disease was acute correction, typically through high tibial osteotomy, as identified in almost half of cases. However, there were substantial variations in the type and location of these osteotomies, making it challenging to determine the optimal method. Hemiepiphysiodesis was reported in 22% of cases, while gradual correction with an external fixator was reported in 18%. Notably, hemiepiphysiodesis exhibited the least favorable outcomes in terms of recurrence (29%), leading the author to suggest that it should no longer be considered as a standalone solution. This is in line with previous systematic reviews about growth modulation in Blount’s disease. Conversely, encouraging results were seen when hemiepiphysiodesis was combined with acute osteotomy, supporting the idea that combined surgical procedures may be beneficial in patients with a high risk of recurrence. However, the small number of cases available for analysis did not fully support this hypothesis.

The investigation into the impact of age at the time of surgery on recurrence aligns with many other authors’ findings, which also identified a significant age threshold, between four and five years, for ITV. Ferriter and Shapiro, in 1987, found that surgery after four and a half years of age was a statistically significant risk factor for recurrence after high tibial osteotomy, reporting a 31% recurrence rate with osteotomy before the age of four and a half, and a recurrence rate of 76% after the age of four and a half [61]. These results were confirmed by Chotigavanichaya et al. in 2002 and by Van Greunen and Firth in 2022, who reported recurrence rates of 46% and 25% before the age of four and of 91% and 67% after the age of four, respectively [73,86]. From the data available for meta-analysis, in ITVs followed up for at least three years, the overall recurrence rate was 5% with treatment before the age of four, compared to 37% after the age of four. This emphasizes the importance of timely and decisive surgical intervention for ITV, to prevent the immediate and high risk of recurrence. Moreover, the risk of recurrence was significantly lower for LOTVs, confirming a milder condition that does not progress once growth stops. However, this bimodal distribution of recurrence risk across various age ranges has significant statistical implications. It has the potential to obscure the primary influences of age at presentation and timing of surgery, making children—particularly those aged between 4 and 11—who are at a higher risk of recurrence, less evident. In our study, the highest recurrence rate was observed in children aged between 4 and 11 who were followed for an appropriate duration. Within this age group, the recurrence rate surpassed 39% on average, and exceeded 60–76% in some reports [61,73]. Remarkably, the minimum follow-up duration in this patient subgroup is a noteworthy variable, as recurrence could potentially go unnoticed in cases with less than 2.5 years of follow-up.

This study also aimed to assess surgical treatment complications beyond deformity recurrence. Across the studies, a consistently low rate of major complications was observed, regardless of the surgical technique applied. This is consistent with previous systematic reviews, underscoring the general safety of surgical management for Blount’s disease. Conversely, minor complications were notably more frequent when gradual correction through external fixators was employed. We contend that, while gradual correction remains a viable and effective option in treating Blount’s disease, it is crucial to pay heightened attention to the emotional and psychosocial impact of external fixator correction on the patient. The time spent in the fixator and the obstacles encountered during treatment are now recognized as relevant and non-negligible complications [87].

Currently, the optimal approach toward balancing stable correction and complication risk likely involves a combination of acute correction via osteotomy and lateral hemiepiphysiodesis of the tibial plateau. This solution appears to yield a sufficiently low recurrence rate and complication risk. Notably, a double osteotomy (medial plate elevation and dome osteotomy) coupled with lateral hemiepiphysiodesis seems to offer the best outcomes, as suggested by some authors [43,66,74]. However, further studies on large series are required to confirm this observation, and should also consider the potential risk of limb shortening and the need for contralateral epiphysiodesis as a precautionary measure to mitigate this effect.

### 4.3. Limitations

Despite the large number of cases and studies included in this systematic review, some limitations must be considered. Firstly, although ITV and LOTV are subgroups with distinctive features and different prognoses, we found significant heterogeneity among authors in how patients with Blount’s disease were classified. Moreover, some authors did not differentiate infantile forms from late-onset ones. This study underscores the need for a standardized and reliable way to classify Blount’s disease.

Secondly, the variability of surgical techniques and instrumentations, which were divided into only six main groups, might show biased results. The AC group, for example, included all fixation techniques, from casting with no fixation, to plates, or even external fixation. Similarly, the GC group included conventional ring fixators, monoaxial devices, and Taylor Spatial Frames. A classification system was essential to condensing over a hundred diverse surgical techniques, occasionally with slight variations, into a handful of coherent subgroups. This allowed for the comparison of the basic principles of surgical treatment for Blount’s disease. However, it is important to note that our classification system was arbitrary and based on our expertise and understanding. Further validation is required to confirm the robustness and statistical validity of our findings. Thirdly, raw data were accessible for only 40% of cases, and only in fewer than half of those were there records of recurrence and follow-up. It is worth noting that many significant and recent studies with large case numbers did not include raw data, potentially introducing additional bias by excluding these extensive datasets from the meta-regression analysis. This issue may partially account for the disparity between the results obtained from the pooled analysis of all articles and the findings derived from the analysis of raw data alone, especially in terms of recurrence rates. However, our observations align with the results derived from the most extensive and well-conducted studies found in the literature, confirming the credibility of our findings [32,50,53,55,60,71,73,74].

## 5. Conclusions

Surgical treatment for Blount’s disease remains a subject of debate, with ongoing controversy regarding the optimal operative approach. Currently, surgical strategies largely depend on the experience and confidence of the individual surgeon with a particular technique. This systematic literature review provided limited evidence to guide the choice of the most effective strategy for managing this condition. However, patients treated before the age of 4.5 years showed the lowest recurrence rate, regardless of the surgical technique used, while children treated between 4.5 and 11 years of age showed a recurrence rate of 39% at a minimum follow-up of 2.5 years. Our study confirmed the need for early diagnosis and timely treatment. Future clinical studies should prioritize the clear differentiation of cases between ITV and LOTV, with emphasis on the former.

## Figures and Tables

**Table 1:** List of the 63 articles selected for systematic review in alphabetical order by first author. Design, level of evidence, nation, quality assessment scores, recurrence rate, and complications are specified. (*)—Fractions represent the number of patients included for analysis among all patients reported by the author(s). CEBM—Oxford Centre for Evidence-Based Medicine (CEBM) criteria; mCMS—modified Coleman Methodology Score; MINORS—Methodology Index for Non-Randomized Studies; FU—follow-up; CS—case series; RCS—retrospective comparative study; GC—gradual correction; AC—acute correction; hE—lateral hemiepiphysiodesis of proximal tibia; hChD—hemichondrodiastasis with external fixator; TSF—Taylor spatial frame; LLD—leg length discrepancy; MAC—multi-axial correction; MPDA—medial plate depression angle; LCP—locking compression plate; TBP—tension band plate.

Paper and Year	Design CEBM	Patients *	Details by Number of Knees	Nation	mCMS	MINORS	Mean FU (Range)	Recurrence	Complications
Alekberov et al. 2003 [15]	CS 4	45/45 24 bilateral	49: metaphyseal tibial osteotomy and GC with Ilizarov 9: metaphyseal tibial osteotomy, acute derotation, and GC with Ilizarov 9: metaphyseal tibial osteotomy and GC with Ilizarov + femoral osteotomy and GC with Ilizarov 2: metaphyseal tibial osteotomy, acute derotation, and GC with Ilizarov + femoral osteotomy and GC with Ilizarov	Russia Turkey	49	10/16	6.5 (2.3–14.8)	9%	16%
Amer and Khanfour 2010 [42]	CS 4	20/20 2 bilateral	22: metaphyseal tibial osteotomy, acute derotation, and GC with Ilizarov	Egypt	26	9/16	2.9 (2.0–4.0)	45%	0%
Andrade and Johnston 2006 [16]	CS 4	24/24 3 bilateral	27: physeal bar resection, metaphyseal tibial osteotomy, and K-wires	USA	63	10/16	3.9 (0.8–7.1)	41%	4%
Assan et al. 2021 [50]	RCS 3b	17/17 7 bilateral	14: proximal tibial lateral epiphysiodesis with plate 10: Rab osteotomy and screws	Benin France	33	12/24	hE 2.0 AC 1.9	hE 0% AC 60%	14%
Bar-On et al. 2008 [27]	CS 4	4/4	4: medial plate acute elevation + metaphyseal osteotomy and GC with TSF	Israel	38	7/16	2.4 (2.0–3.2)	0%	50%
Baraka et al. 2021 [43]	CS 4	19/19 2 bilateral	21: medial plate elevation, metaphyseal tibial dome osteotomy, and K-wires + lateral percutaneous epiphysiodesis	Egypt	62	12/16	5.1 (3.2–8.3)	0%	24%
Beck et al. 1987 [38]	CS 4	3/3	3: physeal bar resection, metaphyseal tibial osteotomy, and pins	USA	19	3/16	1.9 (1.1–2.3)	0%	0%
Blount 1937 [44]	CS 4	6/13 1 bilateral	6: metaphyseal osteotomy and cast 1: medial plate elevation + lateral percutaneous epiphysiodesis and cast	USA	22	6/16	n.d.	AC 33% AC + hE 0%	n.d.
Bushnell et al. 2009 [51]	CS 4	53/53 14 bilateral	45: proximal tibial lateral epiphysiodesis with staples 20: proximal tibial lateral epiphysiodesis with staples + distal femoral lateral epiphysiodesis with staples 2: metaphyseal tibial osteotomy + distal femoral lateral epiphysiodesis with staples	USA	39	6/16	n.d.	40%	4%
Castañeda et al. 2008 [52]	CS 4	21/48 14 bilateral	35: lateral proximal tibial epiphysiodesis	USA	33	8/16	3.0	n.d.	0%
Cherkashin et al. 2015 [45]	CS 4	31/31 2 bilateral	33: metaphyseal tibial osteotomy and GC with TSF or Ilizarov, with or without lengthening	USA	10	5/16	n.d.	21%	100%
Clarke et al. 2009 [53]	RCS 3b	38/38 16 bilateral 4 treated twice	20: metaphyseal tibial osteotomy (and medial plateau elevation if MPDA > 15°) and GC with MAC system + proximal tibial lateral epiphysiodesis 38: metaphyseal tibial osteotomy (and medial plateau elevation if MPDA > 15°) and GC with other external fixators + proximal tibial lateral epiphysiodesis	USA	50	16/24	1.9	24%	100%
Coogan et al. 1996 [54]	CS 4	8/8 4 bilateral	12: metaphyseal tibial osteotomy and GC with Ilizarov (distal AC of valgus of tibia in some cases)	USA	54	8/16	1.9	8%	58%
Danino et al. 2020 [55]	RCS 3b	45/71 10 bilateral	55: proximal tibial lateral epiphysiodesis	Israel Germany USA Austria Canada	38	10/16	2.0 (1.0–4.4)	27%	11%
Doyle et al. 1996 [56]	CS 4	17/17 11 bilateral	13: single proximal tibial osteotomy 13: two or more surgical procedures for proximal tibial osteotomy 2: proximal tibial lateral epiphysiodesis	USA	47	8/16	14.8 (3.2–27.2)	AC 27% hE 50%	AC 12% hE 0%
Eamsobhana et al. 2014 [57]	CS 4	38/38 27 bilateral	65: metaphyseal tibial osteotomy	Thailand	33	10/16	3.0	15%	n.d.
Edwards et al. 2017 [46]	CS 4	7/7 1 bilateral	8: medial plate acute elevation + metaphyseal osteotomy and GC with TSF	UK	42	10/16	4.6 (2.2–9.0)	38%	88%
Eidelman et al. 2008 [47]	CS 4	8/8 2 bilateral	10: metaphyseal osteotomy and GC with TSF (no fibular osteotomy)	Israel	54	10/16	3.6	0%	100%
El Ghafar et al. 2018 [58]	CS 4	13/13 7 bilateral	20: metaphyseal osteotomy, AC, and fixation with Ilizarov	Egypt	26	6/16	1.5	10%	40%
Fahmy and Fathi 2019 [59]	CS 4	13/13 3 bilateral	16: metaphyseal tibial osteotomy and GC with Ilizarov	Egypt	51	10/16	2.0 (1.0–3.0)	0%	63%
Feldman et al. 2006 [60]	RCS 3b	32/32	14: metaphyseal tibial osteotomy, AC and fixation with external fixator 18: metaphyseal tibial osteotomy and GC with TSF (associated lateral tibial epiphysiodesis in 1 case, not separable)	USA	65	16/24	2.0	AC 43% GC 0%	AC 17% GC 21%
Ferriter and Shapiro 1987 [61]	CS 4	25/25 12 bilateral	37: metaphyseal proximal tibial osteotomy (closing wedge, dome, opening wedge), variable types of fixations	USA	25	10/16	4.0 (2.0–9.0)	57%	n.d.
Gordon et al. 2005 [62]	CS 4	15/15 4 bilateral	19: metaphyseal tibial osteotomy and GC with Ilizarov	USA	59	10/16	5.0	0%	100%
Gregosiewicz et al. 1989 [48]	CS 4	10/10 3 bilateral	10: double elevation osteotomy	Poland	50	8/16	8.1 (4.0–14.0)	23%	38%
Hayek et al. 2000 [49]	CS 4	9/9 4 bilateral	13: metaphyseal W osteotomy and AC with K-wires and/or cast	Israel	77	10/16	9.0 (2.5–17.0)	0%	0%
Heflin et al. 2016 [17]	CS 4	17/17 10 bilateral	27: proximal tibial lateral epiphysiodesis	USA	29	8/16	2.5 (0.7–5.8)	15%	22%
Hefny et al. 2010 [18]	CS 4	8/8 4 bilateral	12: double elevation osteotomy combined with GC using Ilizarov frame	Egypt	35	10/16	5.0 (3.0–7.0)	0%	33%
Hollman et al. 2015 [19]	CS 4	17/17 8 bilateral	25: metaphyseal W osteotomy and AC with K-wires and/or cast	Ghana	37	8/16	Short-term	0%	4%
Iliadis et al. 1996 [63]	CS 4	17/17 6 bilateral	23: metaphyseal tibial osteotomy proximal to the tibial tubercle and K-wires	Greece	32	6/16	4.5	17%	9%
Jain et al. 2020 [64]	CS 4	40/40 19 bilateral	61: proximal tibial lateral epiphysiodesis with TBP	USA	51	10/16	3.2 (0.7–9.9)	41%	8%
Janoyer et al. 2007 [20]	CS 4	8/8 1 bilateral	9: medial plate osteotomy and GC with external fixator	France	59	9/16	2.0 (1.2–3.8)	11%	100%
Jones et al. 2003 [21]	CS 4	7/7	7: (step I) medial plate osteotomy, GC (step II) tibial osteotomy, and GC of residual deformity, rotation and LLD	UK	59	9/16	2.4 (1.3–3.7)	50%	100%
Khanfour and El Rosasy 2014 [22]	CS 4	20/20 11 bilateral	30: metaphyseal osteotomy, AC and fixation with mini-Ilizarov 1: metaphyseal osteotomy and GC with Ilizarov	Egypt	47	11/16	AC 5.9 (5.0–7.0) GC 7.0	AC 13% GC 0%	100%
Langenskiöld and Risika 1964 [23]	CS 4	59/65 26 bilateral	85: proximal metaphyseal curved osteotomy and cast	Finland	44	9/16	7.3 (0.5–15.8)	41%	n.d.
Laurencin et al. 1996 [24]	CS 4	8/11	8: lateral closing-wedge metaphyseal osteotomy and plate	USA	45	10/16	8.5 (4.0–13.0)	0%	13%
Liu et al. 2015 [65]	CS 4	12/12 5 bilateral	12: metaphyseal proximal tibia dome osteotomy and AC with K-wires, always with valgizing femoral osteotomy and AC with plate	China	50	8/16	9.0 (3.0–16.0)	8%	0%
Maré et al. 2021 [66]	CS 4	48/48 16 bilateral	50: medial plate elevation, proximal tibial osteotomy, screw fixation, and lateral tibial epiphysiodesis 14: medial plate elevation osteotomy, screw fixation, and lateral tibial epiphysiodesis	South Africa	40	9/16	3.2 (1.0–6.2)	19%	13%
Maré et al. 2022 [25]	CS 4	14/14 4 bilateral	18: proximal tibial lateral epiphysiodesis with TBP	South Africa	34	6/16	2.7 (1.4–5.2)	22%	33%
Martin et al. 1994 [26]	CS 4	7/9 4 bilateral	9: metaphyseal proximal tibial osteotomy and plate fixation 2: metaphyseal proximal tibial osteotomy, proximal lateral tibial epiphysiodesis, and plate fixation	USA	40	5/16	n.d.	AC 11% AC + hE 100%	AC 0% AC + hE 100%
Medbö 1964 [28]	CS 4	17/17 12 bilateral	29: proximal tibial osteotomy, Blount staple fixation, and cast	Norway	29	9/16	9.8 (1.0–17.0)	59%	24%
Miraj et al. 2019 [29]	CS 4	17/17 10 bilateral	27: metaphyseal proximal tibial step-cut V osteotomy and LCP plate fixation	Indonesia	39	7/16	1.0	15%	0%
Mousa 2014 [30]	CS 4	9/9 5 bilateral	14: metaphyseal proximal tibial Chevron osteotomy, wedge transfer, and plate	Egypt	54	9/16	1.0	7%	7%
Nada et al. 2021 [67]	CS 4	11/11	11: medial plate elevation, closing-wedge osteotomy and plate fixation	Egypt	70	10/16	2.2 (1.5–3.0)	n.d.	18%
Ogbemudia et al. 2011 [68]	CS 4	31/31 16 bilateral	47: anteroposterior inverted ‘U’ metaphyseal tibial osteotomy and cast	Nigeria	67	12/16	3.2	n.d.	n.d.
Oto et al. 2012 [31]	CS 4	5/5 1 bilateral	6: lateral proximal tibial epiphysiodesis with TBP	Turkey	59	12/16	2.0 (1.1–2.6)	100%	0%
Oyemade 1981 [69]	CS 4	25/25 15 bilateral	40: metaphyseal proximal tibial wedge osteotomy and cast	Nigeria	28	4/16	n.d.	10%	15%
Pandya et al. 2009 [70]	CS 4	17/17 1 bilateral	18: proximal tibial osteotomy and GC with MAC system	USA	62	10/16	1.7	17%	50%
Park et al. 2005 [71]	CS 4	26/26 7 bilateral	33: lateral proximal tibial epiphysiodesis with stapling	USA	51	10/16	3.7 (2.0–6.8)	33%	18%
Price et al. 1995 [32]	CS 4	25/25 9 bilateral	26: metaphyseal proximal tibial osteotomy, AC, and fixation with monoaxial fixator 4: metaphyseal proximal tibial osteotomy, AC of varus, and GC of LLD with monoaxial fixator 4: GC with hemichondrodiastasis with external fixator	USA	33	9/16	n.d.	AC 15% GC 0% hChD 75%	AC 19% GC 25% hChD 100%
Rab 1988 [33]	CS 4	6/6 1 bilateral	7: Rab osteotomy of proximal tibia and fixation with screws	USA	46	9/16	1.3 (0.8–2.0)	14%	29%
Sachs et al. 2015 [34]	CS 4	22/23 2 bilateral	24: metaphyseal osteotomy and gradual tibial lengthening with TSF	Israel	48	8/16	n.d.	0%	42%
Schoenecker et al. 1992 [35]	CS 4	7/7	3: (stage A) medial plateau elevation, graft, and fixation with pinning or plate (no fixation in 1 patient) + (stage B) metaphyseal proximal tibial osteotomy 3: stage B and, after 7 to 15 months, stage A 1: stage A and, after 7 months, stage B	USA	54	8/16	3.2 (2.0–6.0)	14%	14%
Scott 2012 [36]	CS 4	12/12 6 bilateral	18: lateral proximal tibial epiphysiodesis with TBP	USA	44	9/16	1.6 (0.1–3.1)	11%	28%
Smith et al. 2000 [72]	CS 4	19/19 4 bilateral	23: metaphyseal proximal tibial osteotomy, AC, and fixation with monoaxial external fixator	USA	49	8/16	2.7 (0.5–7.1)	17%	35%
Stanitski et al. 1998 [37]	CS 4	10/14	10: metaphyseal proximal tibial osteotomy and GC with T-Garches fixator	USA	46	8/16	1.4 (0.8–2.6)	20%	40%
Tavares and Molinero 2006 [39]	CS 4	4/5	4: (stage A) medial plate elevation with graft and lateral tibial epiphysiodesis with staples or percutaneous drilling; (stage B) after 3 months, metaphyseal osteotomy and GC with Ilizarov, TSF, or monoaxial fixator	USA	49	10/16	3.3 (3.0–4.0)	0%	0%
Tsibidakis et al. 2018 [40]	CS 4	16/16 8 bilateral	24: metaphyseal proximal tibial osteotomy and GC with TSF	Italy Greece Bulgaria	42	10/16	3.8 (3.0–6.0)	13%	25%
Van Greunen and Firth 2022 [73]	CS 4	44/44 16 bilateral	60: metaphyseal proximal tibial osteotomy and fixation with K-wires	South Africa	33	8/16	2.3 (1.0–6.2)	63%	n.d.
van Huyssteen et al. 2004 [74]	CS 4	24/24 10 bilateral	34: elevating osteotomy, and the remaining tibial varus and internal torsion with an osteotomy just below the apophysis and proximal lateral tibial epiphysiodesis (19 concomitant epiphysiodesis, 15 of them 3 and 12 months after the osteotomy)	South Africa	59	10/16	2.8	3%	9%
Wenger et al. 1984 [41]	CS 4	6/7 2 bilateral	8: corrective osteotomy below growth plate	USA	26	4/16	1.0	0%	n.d.
Westberry et al. 2004 [75]	CS 4	23/23 10 bilateral	21: proximal lateral tibial stapling only 9: proximal lateral drill hemiepiphysiodesis 3: simultaneous proximal lateral tibial stapling and distal lateral femoral stapling	USA	47	6/16	3.1	27%	30%
Wilson et al. 2007 [76]	CS 4	29/29 9 bilateral	38: high tibial osteotomy, AC, and fixation with Ilizarov	USA	39	6/16	2.0	18%	100%
Zein et al. 2021 [77]	CS 4	30/30 2 bilateral	32: AC with minimally invasive osteotomy and simple circular fixation	Egypt	43	10/16	2.1 (2.8–3.8)	0%	34%

**Table 2:** recurrence rate and complication rate among different surgical groups.

Group	Knees (N)	Recurrence (Mean % and C.I. 95%)	I^2^	Minor Complications (Mean % and C.I. 95%)	I^2^	Major Complications (Mean % and C.I. 95%)	I^2^
GC	306 (18%)	7% (1–15%)	0.27	49% (34–64%)	0.57	2% (0–9%)	0.33
GC + hE	76 (5%)	15% (0–40%)	1	74% (37–99%)	0.55	16% (1–41%)	1
AC	788 (47%)	22% (15–29%)	0.34	14% (7–22%)	0.54	6% (2–12%)	0.01
AC + hE	129 (8%)	5% (2–20%)	0.22	0% (0–8%)	0.12	4% (2–17%)	1
hE	369 (22%)	29% (19–40%)	0.11	1% (1–6%)	0.42	4% (0–10%)	0.01
hChD	4 (0.2%)	75% (-)	-	100% (-)	-	0% (-)	-
Total	1672	18% (14–22%) *	0.22 *	18% (12–24%) **	0.60 **	5% (2–8%) **	0.01 **

GC—gradual correction; AC—acute correction; hE—lateral hemiepiphysiodesis of proximal tibia; hChD—hemichondrodiastasis with external fixator; C.I.—confidence interval; I^2^—heterogeneity (*)—data about recurrence were missing in 94 cases (6% of the entire pool). (**)—data about complications were missing in 320 cases (19% of the entire pool).

## Data Availability

Data are available from the corresponding author upon reasonable request.

## Supplementary Materials

The following supporting information can be downloaded at: https://www.mdpi.com/article/10.3390/jcm12206495/s1, Table S1: Preoperative radiographic features evaluated by the authors and selected for raw data analysis, in alphabetical order; Figure S1: Distribution of patients by age at surgery, and sex; Table S2: Results of STATA threshold regression models for follow-up and age at treatment; Table S3: Recurrence rate and complications among patients aged between 4.5 and 11.25 years of age followed for 2.5 or more years; Table S4: Recurrence rate and complications among patients aged below 4 years of age, with follow-up duration specified; Table S5: Recurrence rate and complications among patients aged over 11.25 years of age, with follow-up duration specified.
