# Supplementary material for: Recurrence and Complication Rates of Surgical Treatment for Blount’s Disease in Children: A Systematic Review and Meta-Analysis
Source: J Clin Med. 2023 Oct 12;12(20):6495. doi: 10.3390/jcm12206495 (PMC10607610; doi:10.3390/jcm12206495)
Supplement: Supplementary file 1 [file jcm-12-06495-s001.zip › jcm-2609808-supplementary.pdf]

SUPPLEMETARY MATERIALS

Study	Knees (N)	Age (range)	Langensk.	MADz	MADmm	HKA	aTFA	FC-TS	LDFA	LDTA	MPDA	mMPTA	MDA	PPTA
Alekberov et al., 2003	69	3.2-17.8					69							
Amer et al. 2010	22	8.0-14.0	22			22								
Andrade and Johnson., 2006	27	5.0-10.0	27			27								
Bar-On et al., 2008	4	6.9-9.1	4			4						4		
Baraka et al., 2021	21	8.2-13.6	21				21				21	21	21	21
Beck et al., 1987	4	5.3-9.9	4											
Blount, 1937	7	4.0-14.0												
Cherkashin et al., 2015	33	6.0-18.0	21											
Edwards et al., 2017	8	6.6-10.6	8		8				8		8	8		
Eidelman et al., 2008	10	13.0-17.0			10							10		10
Gregosiewicz et al., 1989	13	6.0-12.0	13				13							
Hayek et al., 2000	13	2.8-5.1	13				13							
Heflin et al., 2016	27	1.8-15.1												
Hefny et al., 2010	12	n.d.	12				12	12			12			
Hollman et al., 2015	25	2.0-9.0	25				25				25		25	
Janoyer et al., 2007	9	7.2-13.5					9				9			
Jones et al., 2003	7	7.1-15.0						7						
Khanfour and El Rosasy, 2014	31	n.d.	31				31							
Langenskiöld et al., 1964	85	2.0-18.5												
Laurencin et al., 1996	8	12.0-16.3												
Maré et al., 2022	18	2.9-11.8	7	18			18				18		18	
Martin et al., 1994	11	3.1-14.3	8				11							
Medbö, 1964	29	3.0-17.0												
Miraj et al., 2019	27	2.0-17.0	27				27						27	
Mousa, 2014	14	14.0-16.0										14		
Oto et al., 2012	6	12.4-14.1			6							6		
Price et al., 1995	34	3.3-16.2	14			31	34				34			
Rab, 1988	7	5.8-13.5												
Sachs et al., 2015	24	12.0-18.0			24					24		24		24
Schoenecker et al., 1992	7	10.2-13.7	7					7			7			
Scott, 2012	18	2.9-8.7				18								
Stanitski et al., 1998	10	11.9-16.1												
Tavares and Molinero, 2006	4	6.2-10.2												
Tsibidakis et al., 2018	24	3.0-14.0					24					24	24	
Wenger et al., 1984	8	12.8-14.3												
**TOTAL**	**676**		**264**	**18**	**48**	**102**	**307**	**26**	**8**	**24**	**134**	**111**	**115**	**55**
			**Langensk.**	**MADz**	**MADmm**	**HKA**	**aTFA**	**FC-TS**	**LDFA**	**LDTA**	**MPDA**	**mMPTA**	**MDA**	**PPTA**
**Correlation with recurrence (*p*-value)**			0.240	0.382	0.499	0.802	0.812	**0.013**	0.071	0.553	0.244	0.244	0.832	0.320

Table S1: preoperative radiographic features evaluated by authors selected for raw data analysis in alphabetical order. N = number; Langensk. = Langenskiöld classification; MADz = mechanichal axis distance (MAD) expressed in zone; MADmm = mechanichal axis distance (MAD) expressed in millimetres; HKA = hip-knee-ankle angle; aTFA = anatomical tibio-femoral angle; FC-TS= angle between femoral condyles and tibial shaft; LDFA = lateral distal femoral angle; LDTA = lateral distal tibial angle; MPDA = medial plate depression angle, between medial tibial plate and lateral tibial plate; mMPTA = mechanical medial proximal tibial angle; MDA = metaphyso-diaphyseal angle or angle of Drennan-Levine; PPTA = posterior proximal tibial angle.

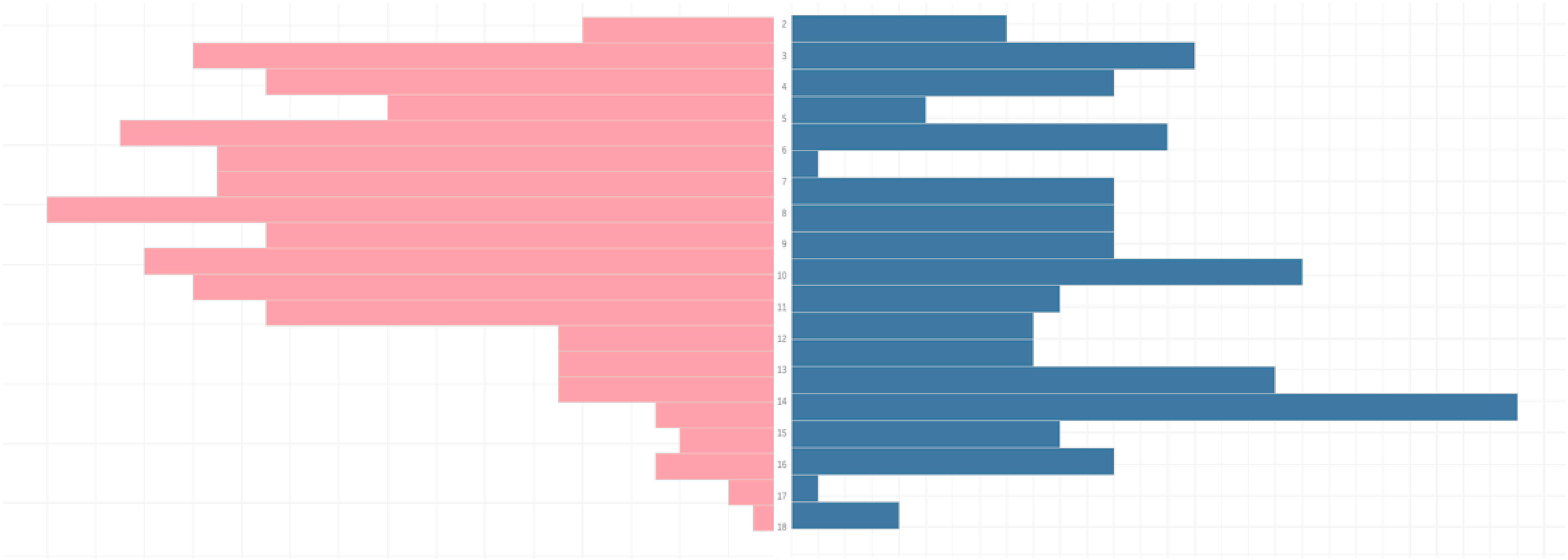

Figure S1: distribution of patients by age at surgery and sex. Females are depicted in pink, males in blue.

**Threshold regression model regions**	**SSR for upper threshold**	**Recurrence % (95% C.I.)**	***p*-value for region**
Follow-up			
<1.7 years	83.8	13% (3–22%)	0.008
1.7–2.5 years	81.6	47% (36–58%)	0.0001
2.5–5.5 years	80.0	17% (9–24%)	0.0001
>5.5 years	-	31% (25–38%)	0.0001
Age at treatment in patients with at least 2.5 years of follow-up			
<4.5 years	63.9	9% (5–18%)	0.039
4.5–11.25 years	67.8	35% (30–41%)	0.0001
>11.25 years	-	5% (0–12%)	0.111

Table S2: results of STATA threshold regression models for follow-up and age at treatment. In the upper part of the table are shown thresholds found for recurrence rate by increasing follow-up. A peak of recurrences emerged before 2.5 years of follow-up, thus this value of follow-up should be sufficient to avoid underestimation of recurrences. In the lower part of the table are shown thresholds found for recurrence rate by varying age at treatment in patients with follow-up of 2.5 years or more.

Authors	Year	Technique	Mean years of age at surgery (range)	Mean follow-up years (range)	Recurrence	Complications (major)
**Acute correction**						
Langenskiöld & Risika	1964	AC: single osteotomy	7.6 (4.5 – 11.0)	8.4 (3.3 – 15.8)	23/42 (55%)	n.d.
Medbö	1964	AC: single osteotomy	7.7 (5.0 – 11.0)	8.7 (3.0 – 13.0)	14/17 (82%)	5/17 (5)
Gregosiewicz et al.	1989	AC: single osteotomy	6.0	9.0	1/1 (100%)	1/1 (1)
Gregosiewicz et al.	1989	AC: MPE with graft + valgizing osteotomy	7.8 (6.0 – 10.0)	8.6 (5.0 – 14.0)	2/10 (20%)	3/10 (1)
Hayek et al.	2000	AC: single osteotomy	4.8 (4.5 – 5.2)	2.5 (2.5 – 2.6)	0/2 (0%)	0/2 (0)
Andrade & Johnston	2006	AC: MPE with cement + valgizing osteotomy	7.1 (5.0 – 10.0)	4.9 (2.5 – 7.1)	12/18 (67%)	n.d.
		**Total AC**	**7.4 (4.5 – 11.0)**	**7.7 (2.5 – 15.8)**	**52/90 (58%)****	**9/30 (7)**
**Acute correction + hemiepiphysiodesis**						
Tavares & Molinero	2006	AC: double osteotomy in two stages + hE	7.9 (6.2 – 9.6)	3.0 (3.0 – 3.0)	0/2 (0%)	0/2 (0)
Baraka et al.	2021	AC: double osteotomy + hE	9.7 (8.2 – 11.1)	5.3 (4.1 – 8.3)	0/16 (0%)	n.d.
		**Total AC + hE**	**9.5 (6.2 – 11.1)**	**5.1 (3.0 – 8.3)**	**0/18 (0%)**	**0/2 (0)**
**Gradual correction**						
Alekberov et al. *	2003	GC	8.4 (5.4 – 11.2)	7.2 (3.5 – 14.8)	4/35 (11%)	2/35 (0)
Jones et al.	2003	GC	8.8 (7.1 – 10.9)	3.4 (2.8 – 3.7)	2/3 (66%)	5/3 (2)
Amer & Khanfour *	2010	GC	9.3 (8.0 – 10.0)	3.3 (3.0 – 4.0)	5/9 (56%)	0/9 (0)
Edwards et al.	2017	GC	9.5 (6.6 – 10.6)	4.9 (2.9 – 9.0)	3/7 (43%)	5/7 (1)
		**Total GC**	**9.0 (5.8 – 12.0)**	**6.1 (2.8 – 14.8)**	**14/54 (26%)**	**12/54 (3)**
**Other techniques**						
Tavares & Molinero	2006	GC + hE	9.5 (8.8 – 10.2)	3.5 (3.0 – 4.0)	0/2 (0%)	0/2 (0)
Janoyer et al.	2007	GC + hE	8.6 (-)	3.8 (-)	0/1 (0%)	1/1 (0)
Heflin et al.	2016	hE	5.4 (5.4 – 5.4)	5.8 (5.8 – 5.8)	0/2 (0%)	0/2 (0)
		**Total other techniques**	**8.3 (5.4 – 11.6)**	**4.2 (3.0 – 5.8)**	**0/5 (0%)**	**1/5 (0)**
		**TOTAL**	**8.1 (4.5 – 11.2)**	**6.7 (2.5 – 15.8)**	**66/169 (39%)**	**22/91 (10)**

Table S3: recurrence rate and complications among patients aged between 4.5 and 11.25 years of age followed for 2.5 or more years. GC = gradual correction; AC = acute correction; hE = lateral hemiepiphysiodesis of proximal tibia; MPE = medial plate elevation. (*) = authors that did not clearly differentiated ITV/LOTV diagnosis. (**) = AC group showed a recurrence rate of 58%, with no statistically significant difference among double osteotomy (50%) and the single osteotomy (61%, p = 0.315).

Authors	Year	Technique	Mean years of age at surgery (range)	Mean follow-up years (range)	Recurrence	Complications (major)
**Acute correction**						
Blount	1937	AC: single osteotomy	4.0 (4.0 – 4.0)	0.5 (0.5 – 0.5)	0/2	0/2 (0)
Langenskiöld & Risika	1964	AC: single osteotomy	3.0 (2.0 – 4.0)	8.3 (1.0 – 13.8)	1/27 (4%)	n.d.
Medbö	1964	AC: single osteotomy	3.6 (3.0 – 4.0)	15.7 (14.0 – 17.0)	2/7 (29%)	1/7 (1)
Price et al.	1995	AC: single osteotomy	3.5 (3.5 – 3.5)	5.0 (5.0 – 5.0)	0/2 (0%)	0/2 (0)
Hayek et al.	2000	AC: single osteotomy	3.3 (2.8 – 4.2)	10.2 (4.6 – 17.0)	0/11 (0%)	2/11 (0)
Miraj et al.	2019	AC: single osteotomy	3.5 (2.0 – 4.0)	1.0 (1.0 – 1.0)	0/4	0/4 (0)
		**Total AC**	**3.3 (2.0 – 4.2)**	**8.7 (0.5 – 17.0)**	**3/53 (7%)**	**3/26 (1)**
**Other techniques**						
Alekberov et al.*	2003	GC	3.3 (3.3 – 3.3)	3.0 (2.9 – 3.3)	0/3 (0%)	1/3 (0)
Heflin et al.	2016	hE	2.0 (1.8 – 2.4)	2.1 (1.2 – 3.6)	1/8 (13%)	1/8 (0)
		**Total other techniques**	**2.3 (1.8 – 3.3)**	**2.4 (1.2 – 3.6)**	**1/11 (9%)**	**2/11 (0)**
		**TOTAL**	**3.1 (1.8 – 4.2)**	**7.6 (0.5 – 17.0)**	**4/64 (6%)**	**5/37 (1)**

Table S4: recurrence rate and complications among patients aged below 4 years of age, with follow-up duration specified. GC = gradual correction; AC = acute correction; hE = lateral hemiepiphysiodesis of proximal tibia; EF = external fixator. (*) = authors that did not clearly differentiated ITV/LOTV diagnosis.

Authors	Year	Technique	Mean years of age at surgery (range)	Mean follow-up years (range)	Recurrence	Complications (major)
**Acute correction**						
Blount	1937	AC: single osteotomy	13.3 (12.5 – 14.0)	2.0 (2.0 – 2.0)	1/2 (50%)	0/2 (0)
Langenskiöld & Risika	1964	AC: single osteotomy	15.1 (12.5 – 18.5)	4.2 (0.5 – 11.8)	2/4 (50%)	n.d.
Medbö	1964	AC: single osteotomy	14.5 (12.0 – 17.0)	4.0 (1.0 – 7.0)	0/2 (0%)	0/2 (0)
Gregosiewicz et al.	1989	AC: MPE with graft + valgizing osteotomy	12.0 (12.0 – 12.0)	5.0 (4.0 – 6.0)	0/2 (0%)	1/2 (0)
Mousa et al.	2014	AC: local wedge transfer osteotomy	14.9 (14.0 – 16.0)	1.0 (1.0 – 1.0)	1/14 (7%)	1/14 (0)
Miraj et al.	2019	AC: single osteotomy	15.3 (12.0 – 17.0)	1.0 (1.0 – 1.0)	0/3 (0%)	0/3 (0)
		**Total AC**	**14.6 (12.0 – 18.5)**	**2.1 (0.5 – 11.8)**	**4/27 (15%)**	**2/23 (0)**
**Acute correction + hemiepiphysiodesis**						
Blount	1937	AC: single osteotomy + hE	11.5 (-)	2.0 (-)	0/1	n.d.
Baraka et al.	2021	AC: double osteotomy + hE	12.6 (11.6 – 13.6)	3.7 (3.2 – 4.5)	0/5 (0%)	n.d.
		**Total AC + hE**	**12.4 (11.5 – 13.6)**	**3.5 (2.0 – 2.5)**	**0/6 (0%)**	**n.d.**
**Gradual correction**						
Stanitski et al.	1998	GC	13.6 (11.9 – 16.1)	1.5 (0.8 – 2.6)	2/10 (20%)	4/10 (1)
Alekberov et al.*	2003	GC	14.1 (11.4 – 17.8)	6.4 (2.3 – 12.8)	2/29 (7%)	8/29 (0)
Jones et al.	2003	GC	13.5 (12.0 – 15.0)	1.9 (1.7 – 2.2)	1/2 (50%)	3/2 (1)
Eidelman et al.	2008	GC	14.7 (13.0 – 17.0)	3.6 (3.6 – 3.6)	0/10 (0%)	10/10 (1)
Amer & Khanfour*	2010	GC	12.7 (12.0 – 14.0)	2.8 (2.0 – 4.0)	3/10 (30%)	0/6 (0)
		**Total GC**	**13.8 (11.4 – 17.8)**	**4.4 (0.8 – 12.8)**	**8/61 (13%)**	**25/61 (3)**
**Gradual correction + hemiepiphysiodesis**						
Jones et al.	2003	GC + hE	11.4 (-)	1.8 (-)	0/1 (0%)	1/1 (0)
Janoyer et al.	2007	GC + hE	12.6 (11.6 – 13.5)	2.6 (1.8 – 3.3)	1/2 (50%)	2/2 (0)
		**Total GC + hE**	**12.2 (11.4 – 13.5)**	**2.3 (1.8 – 3.3)**	**1/3 (33%)**	**3/3 (0)**
**Hemiepiphysiodesis**						
Oto et al.	2012	hE	13.0 (12.4 – 14.8)	2.0 (1.1 – 2.6)	6/6 (100%)	0/6 (0)
Heflin et al.	2016	hE	13.9 (12.7 – 15.1)	2.2 (0.7 – 4.4)	0/13 (0%)	4/13 (0)
		**Total hE**	**13.6 (12.4 – 15.1)**	**2.1 (0.7 – 4.4)**	**6/19 (32%)**	**4/19 (0)**
		**TOTAL**	**13.9 (11.4 – 18.5)**	**3.4 (0.5 – 12.8)**	**19/116 (16%)**	**34/106 (3)**

Table S5: recurrence rate and complications among patients aged beyond 11.25 years of age, with follow-up duration specified. GC = gradual correction; AC = acute correction; hE = lateral hemiepiphysiodesis of proximal tibia; MPE = medial plate elevation. (*) = authors that did not clearly differentiated ITV/LOT1V diagnosis.
